# A Rat Model of Cocaine-Alcohol Polysubstance Use Reveals Altered Cocaine Seeking and Glutamate Levels in the Nucleus Accumbens

**DOI:** 10.3389/fnins.2020.00877

**Published:** 2020-08-18

**Authors:** Bethany A. Stennett, Lori A. Knackstedt

**Affiliations:** ^1^Psychology Department, University of Florida, Gainesville, FL, United States; ^2^Center for Addiction Research, University of Florida, Gainesville, FL, United States

**Keywords:** relapse, addiction, ethanol, cocaine use disorder, polydrug

## Abstract

Preclinical models of cocaine use disorder are widely utilized to identify neuroadaptations underlying cocaine seeking and to screen medications to reduce seeking. However, while the majority of cocaine users engage in poly-substance use (PSU), a minority of preclinical studies employ PSU models. We previously reported that when rats consume alcohol after daily intravenous cocaine self-administration, nucleus accumbens (NA) core basal glutamate levels are reduced below those of rats that consumed only cocaine, and do not increase during cue + cocaine-primed reinstatement of cocaine-seeking. Here we used the same model of sequential cocaine and alcohol self-administration to test the hypothesis that a similar pattern of glutamate changes would be observed in the NA core prior to and during a cocaine-primed reinstatement test. Rats underwent intravenous cocaine self-administration followed by access to unsweetened alcohol in the home cage for 12 days. Rats underwent a minimum of 12 daily extinction sessions prior to a cocaine-primed reinstatement test conducted during microdialysis procedures. Contrary to our previous work using the same model, here we found that access to alcohol increased cocaine intake and increased responding during early extinction training. We found that as in our previous work, cocaine + alcohol-consuming rats displayed basal glutamate levels below those of rats that self-administered only cocaine. During the cocaine-primed reinstatement test, rats that consumed only cocaine displayed increased glutamate efflux in the NA core while those that consumed cocaine + alcohol did not. These results indicate that preclinical models of PSU should be utilized to develop experimental therapeutics for the reduction of cocaine seeking.

## Introduction

Cocaine use disorder (CUD) remains a problem in the US today, with ~1 million Americans currently meeting DSM-V criteria for CUD (Center for Behavioral Health Statistics and Quality, 2015). Animal models of CUD, such as the operant self-administration model, have consistently found dysregulation of glutamate homeostasis in the nucleus accumbens (NA) core after cocaine self-administration in outbred Sprague-Dawley rats. Glutamate homeostasis refers to the balance between glutamate release and reuptake. This dysregulation includes a decrease in the glutamate transporter GLT-1 expression and function in the NA core and increased synaptic glutamate during reinstatement of cocaine-seeking, regardless of the stimuli priming such reinstatement (e.g., cue, context, cocaine-prime) (McFarland et al., 2003; Knackstedt et al., 2010; Fischer et al., 2013; LaCrosse et al., 2016; Smith et al., 2017; Siemsen et al., 2020). Basal, extra-synaptic glutamate levels are reduced in the NA core at the time of reinstatement testing, caused by reduction in function of the glutamate antiporter system xc- and protein expression of its catalytic subunit xCT (Baker et al., 2003; Knackstedt et al., 2010). Based on such adaptations, medications that restore glutamate homeostasis hold potential for the reduction of cocaine relapse. For example, subchronic treatment with the β-lactam antibiotic ceftriaxone *reliably* attenuates the reinstatement of cocaine-seeking while increasing NA core GLT-1 and xCT protein expression and function, increasing basal extra-synaptic glutamate levels, and preventing the increase in glutamate efflux that accompanies reinstatement (Sari et al., 2009; Knackstedt et al., 2010; Sondheimer and Knackstedt, 2011; Trantham-Davidson et al., 2012; Fischer et al., 2013; Bechard et al., 2018).

However, such cocaine and ceftriaxone-induced effects identified in rodents may not translate into the clinic, as 50–90% of cocaine users also use alcohol (Grant and Harford, 1990; Rounsaville et al., 1991; Anthony et al., 1994; Brookoff et al., 1996; Kedia et al., 2007; Liu et al., 2018). Alcohol consumption in outbred Sprague-Dawley rats leads to increased NA core basal glutamate levels during early abstinence, with no changes in GLT-1 and xCT expression, and ceftriaxone is ineffective at changing GLT-1 expression after alcohol (Pati et al., 2016; Stennett et al., 2017). Thus, the combination of cocaine and alcohol likely leads to unique NA core glutamate adaptations differing from that of cocaine alone. Indeed, we previously found that in a rat model of sequential cocaine and alcohol consumption, where intravenous cocaine self-administration occurred in the operant chamber and was followed by alcohol access in the home cage, different NA core neuroadaptations were present after cocaine + alcohol consumption (Stennett et al., 2020). Specifically, we found that after cocaine + alcohol, NA core GLT-1 expression was increased relative to both drug-naïve controls and rats that consumed only cocaine. In rats that consumed both cocaine + alcohol, NA core basal glutamate was reduced prior to a reinstatement test relative to rats that consumed only cocaine. During a cue + cocaine-primed reinstatement test, NA core glutamate efflux increased in rats that consumed cocaine alone, but not those with a history of both cocaine and alcohol consumption, despite equivalent levels of cocaine-seeking during the reinstatement test. Finally, ceftriaxone was ineffective at attenuating cue, cocaine, and cue + cocaine-primed reinstatement of cocaine seeking in rats with a history of both cocaine and alcohol consumption (Stennett et al., 2020).

Here we sought to extend our previous findings, testing whether NA core glutamate efflux is increased during a cocaine-primed reinstatement test in rats with a history of cocaine + alcohol consumption. This is important because many publications have used the cocaine-primed reinstatement model to investigate neuroadaptations driving relapse to cocaine seeking. Because we previously found that ceftriaxone was unable to attenuate cocaine-primed reinstatement in rats with a history of both cocaine and alcohol consumption, we hypothesize that basal and reinstatement-associated glutamate levels in the NA core will differ between cocaine self-administering rats that do and do not consume alcohol.

## Materials and Methods

### Animals

Male Sprague-Dawley rats (*n* = 20) were acquired from Charles Rivers Laboratories (Raleigh, NC, United States). Upon arrival, they were housed in a temperature- and humidity-controlled vivarium. A reverse 12 h light cycle was used with lights off at 7 a.m. Rats received 20 g/day chow and water *ad libitum*. All procedures were approved by University of Florida’s Institutional Animal Care and Use Committee and were in accordance with National Institutes of Health Guide for the Care and Use of Laboratory Animals.

### Drugs

Cocaine hydrochloride was obtained through the NIDA Drug Supply Program (Research Triangle Institute, NC, United States). Cocaine was dissolved in 0.9% physiological saline for intravenous (i.v.) self-administration and intraperitoneal (i.p.) injection.

### Intermittent Access to Alcohol (IAA)

**FIGURE 1:**
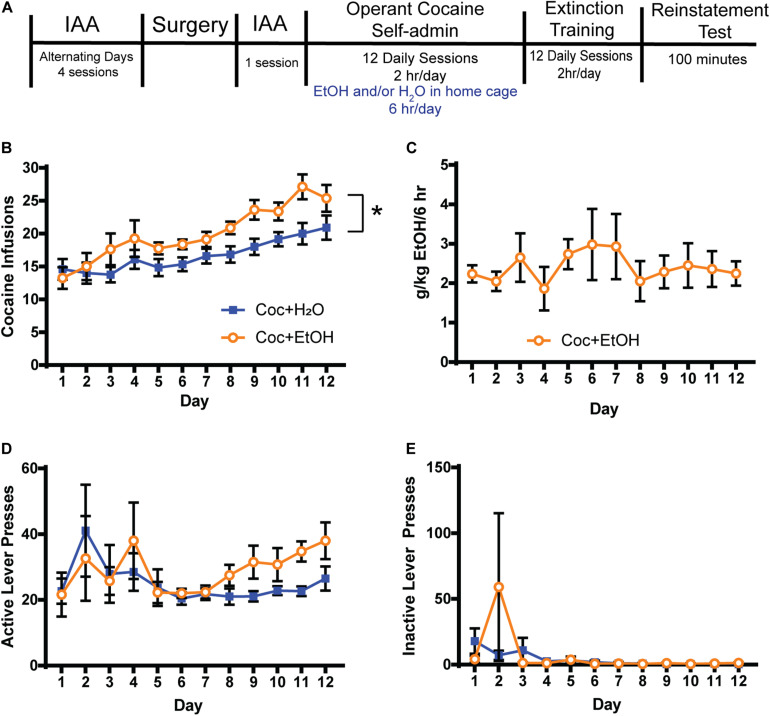
Access to alcohol increases cocaine intake. **(A)** Timeline. Rats were trained to consume alcohol in the home cage using the Intermittent Access to Alcohol (IAA) paradigm prior to beginning operant cocaine self-administration sessions. Access to alcohol and water or water alone was provided for 6 h in the home cage after daily cocaine sessions. Extinction training was conducted for at least 12 days and until criteria was met, at which time a cocaine-primed reinstatement test was conducted. **(B)** A main effect of Liquid [*F*_(1, 18)_ = 5.841, *p* = 0.0265] but no Liquid x Time interaction was found for the number of cocaine infusions attained. Thus, consuming alcohol after cocaine self-administration increases the motivation to consume cocaine on the following day. A main effect of Time was found [*F*_(11, 198)_ = 13.13, *p* = 0.0001], indicating that both groups similarly escalated cocaine intake over the 12 days. **(C)** Alcohol (g/kg) consumed during the 6 h following each cocaine self-administration session. **(D)** Active lever presses did not differ between groups during self-administration. **(E)** Inactive lever pressing was low during self-administration and did not differ between groups. Coc + H_2_O *n* = 12; Coc + EtOH *n* = 8.

Rats (*n* = 8) were provided 24 h access to unsweetened ethanol on alternating days for 4 sessions prior to surgery and one session following surgery (see timeline in Figure 1A). Rats were weighed and then presented with ethanol (20% v/v) and water bottles (2-bottle choice) in the homecage within the first hour of the dark cycle. Food-restriction began the night prior to the first alcohol presentation, and rats were given their daily allotment of food at the same time as the alcohol bottles. Rats that would later have only water available after cocaine (*n* = 12) were only given water during this time.

### Surgery

Rats were anesthetized with ketamine (87.5 mg/kg, IP) and xylazine (5 mg/kg, IP). A catheter made from SILASTIC silicon tubing (ID 0.51 mm, OD 0.94 mm, Dow Corning, Midland, MI, United States) was implanted in the jugular vein and secured with suture thread. The catheter exited an incision on the back where it attached to a stainless-steel cannula inside a rubber harness (Instech, Plymouth Meeting, PA, United States). Rats were next placed into a stereotaxic frame (Stoelting, Wood Dale, IL, United States). A unilateral 22 gauge stainless guide cannula (Synaptech, Marquette, MI, United States) was implanted 2 mm above the NA core according to the coordinates: AP + 1.2 mm, ML ± 1.6 mm, DV −5.5 mm (Paxinos and Watson, 2006). Rats were administered the analgesic ketorolac (2 mg/kg, i.p.) on the day of surgery and for 3 days post-surgery. Catheter patency was maintained with heparinized saline (100 U/mL) daily and periodically verified with methohexital sodium (10 mg/mL i.v.; Eli Lilly, Indianapolis, IN, United States).

### Cocaine and Alcohol Self-Administration

Rats self-administered cocaine in a standard two-lever operant chamber (Med Associates, St. Albans, VT, United States), on an FR-1 schedule of reinforcement, whereby one press on the “active” lever resulted in cocaine (1.05 mg/kg/infusion) and cue-presentation (2900 Hz tone and stimulus light). Presses on the “inactive” lever had no programmed consequences but were recorded. Cocaine self-administration sessions occurred between the hours of 8–10 a.m. daily. Immediately following each 2 h self-administration session, rats were returned to their home cage where they received access to water alone or both water and unsweetened alcohol (20% v/v) for 6 h. Self-administration continued for 12 days, after which rats experienced daily 2 h extinction training, during which time presses on the previously active lever did not deliver drug or cues. No alcohol was available during this time. The criteria for successful extinction was that presses on the previously active lever were less than 50% of the last day of SA for two consecutive days. After completing a minimum of 12 extinction sessions and meeting such criteria, rats underwent cocaine-primed reinstatement testing while undergoing microdialysis procedures as described below. For the cocaine-primed reinstatement test, a 10 mg/kg priming dose of cocaine was administered, and rats were placed into the self-administration chamber with levers extended for 2 h. No tone or drug was delivered following presses of the previously active lever.

### Microdialysis and HPLC for Glutamate

On the night prior to the reinstatement test, microdialysis probes were implanted (2 mm cuprophane membranes, 0.36 mm outer diameter; Synaptech, Marquette, MI, United States) and rats spent the night in their home cage adjacent to operant chambers. Probes were perfused overnight with artificial cerebrospinal fluid (aCSF) containing (125 mM NaCl, 2.5 mM KCL, 1 mM MgCl_2_6H_2_O, 5 mM D-glucose, 1.2 mM CaCl_2_H_2_O, 0.75 mL 10 x phosphate buffered saline, pH-7.3–7.5) at a flow rate of 0.2 μl/min. The next morning, the flow rate was increased to 2.0 μl/min for 2 h. Next, rats were placed into the operant chambers and baseline samples were collected every 10 min for 1 h. Rats then received a cocaine injection (10 mg/kg IP) and levers were extended for a 100 min cocaine-primed reinstatement test, during which presses on the active lever did not yield cues or drug. Samples continued to be collected every 10 min for the duration of the reinstatement test.

Glutamate concentrations were determined via isocratic high-performance liquid chromatography with electrochemical detection (HPLC-ED; Thermo Scientific., Waltham, MA, United States). O-pthalaldehyde (Sigma-Aldrich, St. Louis, MO, United States) was added to samples for derivatization using an autosampler (Thermo Scientific, Inc.) immediately before injection onto a CAPLCELL PAK C18 column (5 μm, 2.0 mm I.D. X 50 mm; Shiseido Inc., Tokyo, Japan). The mobile phase consisted of 100 mM Na_2_HPO_4_, 16% (vol/vol) methanol, and 2.5% (vol/vol) acetonitrile (pH = 6). Glutamate concentrations in the experimental samples were determine by comparing computer-integrated peak areas of samples with those of l-glutamate standards using a 5-point calibration curve (10, 5, 2.5, 1.25, and 0.625 μM).

### Statistical Analyses

Data were analyzed using GraphPad Prism (version 7, GraphPad Software, La Jolla, CA, United States). Behavioral measures and glutamate concentrations were compared using (RM) Analysis of Variance (ANOVA)s with Time as the within-subject factor and Liquid (alcohol/water) as between-subject factors. To assess reinstatement, the average number of lever presses during the last two extinction sessions were averaged and compared with lever pressing during the test. Significant main effects and/or interactions were followed by Tukey’s *post hoc* analyses. AUC glutamate was calculated according to (Chefer et al., 2011), and the AUC during the cocaine-primed reinstatement test was compared between groups using an un-paired t-test. Pearson r tests explored relationships between active lever presses during the reinstatement test, total alcohol consumed, total cocaine consumed, and glutamate levels (AUC) during reinstatement relative to baseline.

## Results

For the eight rats that self-administered cocaine followed by alcohol, mean total alcohol intake during the 5 IAA sessions prior to self-administration was 21.64 ± 2.97 g/kg. There was a main effect of Liquid on the number of cocaine infusions attained during self-administration [*F*_(1, 18)_ = 5.841, *p* = 0.0265; Figure 1B]. No Liquid × Time interaction was found for the number of cocaine infusions attained. A main effect of Time was found [*F*_(11, 198)_ = 13.13, *p* < 0.0001], indicating that both groups similarly escalated cocaine intake over the 12 days. Alcohol intake (g/kg) is depicted in Figure 1C. There were no effects of either Liquid or Time on active (Figure 1D) or inactive lever presses (Figure 1E) during self-administration.

**FIGURE 2:**
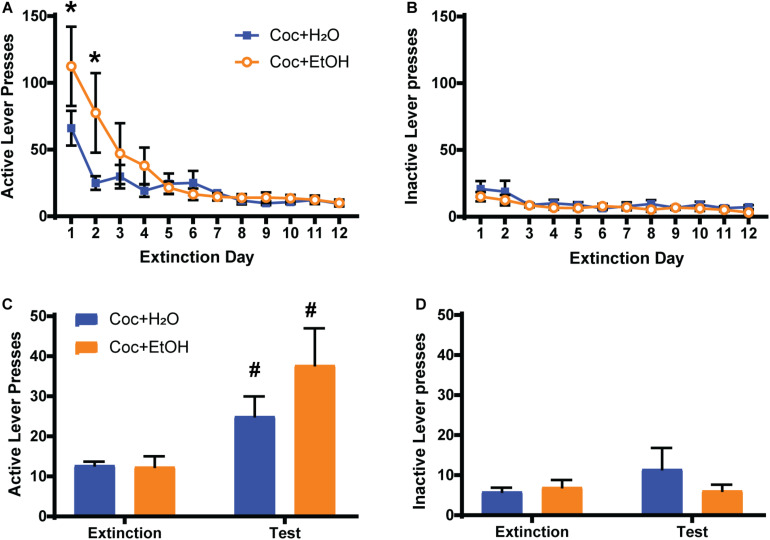
Sequential cocaine and alcohol self-administration increases seeking during extinction training. **(A)** A Liquid × Time interaction was found for active lever presses during extinction training [*F*_(11, 198)_ = 2.469, *p* = 0.0064], with the Coc + EtOH group displaying greater presses on the previously active lever during the first 2 days of extinction. A main effect of Time was also detected [*F*_(11, 198)_ = 13.76, *p* < 0.0001], as both groups decreased lever pressing over the course of extinction training. **(B)** Inactive lever presses were low and did not differ between groups during extinction training. **(C)** A main effect of Time [*F*_(1, 18)_ = 14.45, *p* = 0.0013] was found for active lever presses during the cocaine-primed reinstatement test. Both the Coc + EtOH and the Coc + H_2_O groups reinstated cocaine-seeking. **(D)**. Inactive lever pressing remained low and did not differ between groups during the reinstatement test. *p < 0.05 comparing Coc + EtOH to Coc + H_2_O; ^#^*p* < 0.05 comparing extinction to test.

A Liquid × Time interaction was found for active lever presses during extinction training [*F*_(11, 198)_ = 2.469, *p* = 0.0064], with the Coc + EtOH group displaying greater presses on the previously active lever during the first 2 days of extinction (Figure 2A). A main effect of Time was also detected [*F*_(11, 198)_ = 13.76, *p* < 0.0001], as both groups decreased lever pressing over the course of extinction training. A main effect of Time was found for inactive lever presses during extinction [*F*_(11, 198)_ = 3.864, *p* < 0.0001; Figure 2B]. No main effect of Liquid was found. A main effect of Time [*F*_(1, 18)_ = 14.45, *p* = 0.0013] was found for active lever presses during the cocaine-primed reinstatement test, but no effect of Liquid or Liquid × Time interaction (Figure 2C). Both the Coc + EtOH (*p* = 0.0373) and the Coc + H_2_O (*p* = 0.0258) groups reinstated cocaine-seeking (demonstrated by a significant difference between lever pressing during extinction and test). There were no main effects or interaction for inactive lever pressing during the reinstatement test (Figure 2D). Thus, a history of alcohol consumption did not alter cocaine-primed reinstatement, which is evidenced by increased presses on the previously active lever (but not inactive lever) when comparing extinction to test.

**FIGURE 3:**
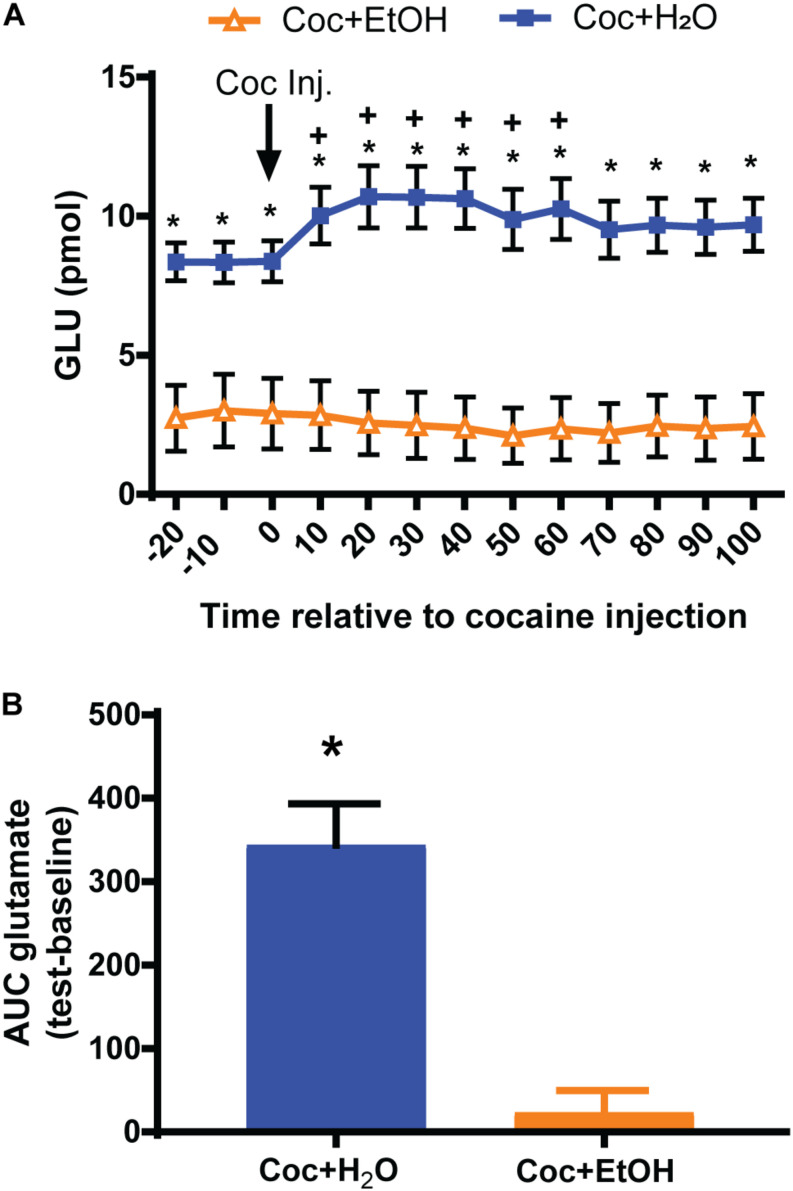
Nucleus accumbens core glutamate efflux prior to and during a cocaine-primed reinstatement test. **(A)** A Liquid × Time interaction [*F*_(12, 216)_ = 6.363, *p* < 0.0001] was found for glutamate levels prior to and during the reinstatement test. Rats which consumed Coc + EtOH displayed reduced baseline glutamate levels relative to the Coc + H_2_O condition. Only the Coc + H_2_O group increased glutamate levels from baseline to test. *p < 0.05 comparing Coc + EtOH to Coc + H_2_O. ^+^*p* < 0.05 compared to own baseline. **(B)** Computing the AUC glutamate levels during baseline and subtracting it from levels during the test reveals a significant difference between groups [*t*_(18)_ = 4.927, *p* < 0.0001].

A significant Liquid × Time interaction was found for glutamate content [*F*_(12, 216)_ = 6.363, *p* = 0.0001; Figure 3A] prior to and during the reinstatement test. *Post hoc* analyses revealed several significant between-group and within-group comparisons. The most notable is that rats which had consumed Coc + H_2_O displayed increased glutamate efflux during the cocaine-primed reinstatement test relative to both baseline values and to the Coc + EtOH group. Rats in the Coc + EtOH group did not display the increase in glutamate efflux characteristic of cocaine-primed reinstatement, as seen in the Coc + H_2_O group. Finally, *post hoc* tests found that rats which consumed Coc + EtOH displayed reduced baseline glutamate levels relative to the Coc + H_2_O condition. In order to conduct correlations with glutamate release during the reinstatement test and other dependent variables, we calculated the AUC glutamate during the test. These values differed between groups [*t*_(18)_ = 4.927, *p* < 0.0001; Figure 3B]. Total alcohol intake correlated with AUC glutamate during the test (*r* = -0.6684, *p* = 0.0013), but not with active lever pressing during Day 1 of extinction or during the reinstatement test. AUC glutamate did not correlate with active lever presses during relapse.

## Discussion

Here we used a model of cocaine and alcohol polysubstance use in which rats voluntarily self-administered cocaine in the operant chamber followed immediately by unsweetened alcohol in the home cage, finding that access to alcohol increased cocaine intake. In our previous work with this model that utilized three cohorts of rats in three separate experiments, access to alcohol after daily cocaine self-administration did not alter cocaine intake, and vice versa, in any of the rat cohorts (Stennett et al., 2020). As the present work was conducted in an identical manner by the same personnel during the same time frame, methodological differences do not explain the discrepant findings. While we did not assess BALs here, in our previous work with this model, rats consumed 2–5 g/kg alcohol in 6 h with an average BAL of 51 mg% attained in the first 2 h of drinking. The amount of alcohol consumed by rats in the present study fell within the range in our previous study, and thus the increase in cocaine intake here did not stem from increased alcohol intake *per se*.

Few studies explore the effect of cocaine and alcohol administration on each other’s consumption. We previously reported that rats given a single non-contingent cocaine infusion (1 mg/kg i.v.) display greater consumption of a sweetened 8% alcohol solution relative to rats receiving saline infusions, over the course of 20 days of testing (Knackstedt et al., 2006). However, rats given the opportunity to traverse an alley to self-administer a single infusion of cocaine (1.0 mg/kg i.v.) do not consume more sweetened 4 or 8% alcohol compared to rats receiving i.v. saline (Knackstedt and Ettenberg, 2005). In rhesus monkeys, daily alcohol access 4 h after cocaine self-administration increases the self-administration of lower doses of cocaine, but not higher doses, and alcohol intake is unaffected by cocaine (Czoty, 2015). Thus, at present, the limited publications on this topic indicate that only non-contingently administered i.v. cocaine increases subsequent alcohol intake in a sequential model where cocaine precedes alcohol access. However, access to alcohol following daily i.v. cocaine self-administration sessions can increase cocaine intake or have no effect. Additional studies using concurrent cocaine-alcohol PSU models are needed to determine whether such increases in cocaine intake are consistently observed. At this time, we propose that genotypic variation in the outbred Sprague-Dawley strain of rats may be the source of discrepant findings. It is also of interest whether human cocaine users report greater consumption of cocaine when consuming alcohol concurrently, however, data on this topic is absent from the literature.

Here we also found that cocaine + alcohol rats displayed increased responding on the previously active lever during the first 2 days of extinction training, despite equivalent responding on this lever during cocaine self-administration. This is additional evidence for enhanced motivation to seek cocaine after cocaine-alcohol PSU, although it could also be an indication of impairment in extinction learning. Future work should use other operant schedules, such as progressive ratio schedules, to investigate this further. A similar increase in responding on the cocaine lever during early extinction training is observed in rats displaying long term anxiety following a single exposure to a predator stressor, a comorbid condition in which the effects of ceftriaxone on the reinstatement of cocaine seeking are also blunted relative to rats with only cocaine self-administration experience as they are in this cocaine-alcohol PSU model (Schwendt et al., 2018; Stennett et al., 2020). Thus, in cases of enhanced motivation to seek cocaine, ceftriaxone may be less effective at reducing such seeking.

As relapse to drug seeking after periods of drug abstinence is a barrier to recovery from CUD, understanding the neurobiology of relapse is essential. The reinstatement of cocaine-seeking has consistently been found to be accompanied by glutamate efflux in the NA core when reinstatement is “primed” by cocaine itself (McFarland et al., 2003; Trantham-Davidson et al., 2012; Lutgen et al., 2014; Logan et al., 2018). Such glutamate release is synaptic, and primarily arises from the PFC-NA core projection (McFarland et al., 2003). Cue-, context- and cue + cocaine-primed reinstatement are also accompanied by glutamate efflux in the NA core (LaCrosse et al., 2016; Smith et al., 2017; Stennett et al., 2020). Thus, it is notable that here, as in our previous work using a cue + cocaine-prime (Stennett et al., 2020), rats with a history of both cocaine and alcohol consumption did not show elevated glutamate in the NA core during reinstatement of cocaine-seeking. This pattern is observed despite equivalent reinstatement between groups consuming alcohol and water. In our previous work with this model, as in the present results, the change in glutamate efflux during the reinstatement test (AUC) negatively correlated with the total amount of alcohol consumed. These results strongly imply that the neurobiology underlying the reinstatement of cocaine-seeking is altered by co-consumption of alcohol. We previously found that cue + cocaine-primed reinstatement did not differ between rats that consumed cocaine alone and cocaine + alcohol. However, despite equivalent reinstatement, reinstatement-induced Fos expression patterns differed, with cocaine + alcohol rats showing reduced Fos in the NA core (in line with lack of increased glutamate efflux during reinstatement) and increased Fos in the BLA. No group differences in Fos expression were found in the NA shell. Thus, it is possible that glutamate or dopamine release in brain regions other than the NA core (e.g., BLA-NA shell) mediate the reinstatement of cocaine-seeking in rats consuming both cocaine and alcohol.

Another notable finding of the present work is that cocaine + alcohol rats displayed reduced glutamate levels prior the reinstatement test, relative to cocaine + water rats, in agreement with our previous work (Stennett et al., 2020). As basal extrasynaptic glutamate levels in the NA core influence synaptic plasticity, and reduction in such levels increase the expression and function of post-synaptic AMPA receptors that drive reinstatement (Conrad et al., 2008; Sun and Wolf, 2009; LaCrosse et al., 2017), this effect has implications for cocaine-seeking. Basal extrasynaptic glutamate levels in the NA core are largely regulated by system xc^–^, which exchanges extracellular cystine for intracellular glutamate. Future work should examine whether reductions in NA core basal glutamate levels stem from reduced system xc^–^ function, as they do following cocaine self-administration alone (Baker et al., 2003). We have shown that in the same concurrent PSU model employed here, rats consuming both cocaine + alcohol have increased surface GLT-1 expression in the NA core relative to rats consuming each drug alone, when assessed 14–17 days after the last self-administration session (Stennett et al., 2020). This finding may contribute to the reduced baseline glutamate levels observed in the cocaine + alcohol group in our previous report and in the present data set. It is worth noting that outbred Sprague-Dawley rats show no changes in NA core GLT-1 or xCT expression following alcohol consumption (Pati et al., 2016; Stennett et al., 2020), and thus display non-additive effects of cocaine + alcohol on GLT-1 expression. However, the inbred alcohol-preferring “P” rats display reduced NA core GLT-1 and xCT following 7 weeks of continuous access to alcohol in the homecage (Das et al., 2015; Hakami et al., 2016), indicating a genetic influence on the ability of alcohol to alter GLT-1 expression. Thus, it would be of interest to examine the same dependent variables following concurrent cocaine-alcohol self-administration in “P” rats to determine the role of GLT-1 and xCT in altering baseline and reinstatement-evoked glutamate release in the NA core. Finally, our concurrent cocaine + alcohol self-administration model employs limited access (2 h/day) to cocaine. While length of daily access to cocaine does not influence the role or magnitude of NA core glutamate efflux during reinstatement of cocaine-seeking (Lutgen et al., 2014), utilizing other cocaine self-administration conditions (e.g., extended or intermittent access) in combination with alcohol access will also be an important future direction and will possibly uncover distinct neuroadaptations.

## Conclusion

Taken together with previous work on this topic, sequential cocaine and alcohol consumption has the potential to increase intake and cocaine-seeking, and alters the neurobiology underlying the reinstatement of cocaine-seeking. Potential mechanisms underlying reinstatement of cocaine-seeking following cocaine + alcohol PSU is currently unknown and should be a focus of future research in order to develop pharmacotherapeutics for reducing cocaine seeking in cocaine + alcohol polysubstance users. Future work should also seek to identify the molecular mechanisms underlying the altered basal and reinstatement-evoked glutamate in the cocaine + alcohol condition. Candidates for such regulation include system xc^–^, the mGlu2/3 glutamate receptor, and GLT-1. We previously reported that cocaine + alcohol rats displayed greater surface GLT-1 expression relative to cocaine + water rats 14–17 days after cessation of self-administration (Stennett et al., 2020), and similar investigations of system xc^–^ and mGlu2/3 should be conducted.

## Data Availability Statement

The raw data supporting the conclusions of this article will be made available by the authors, without undue reservation, to any qualified researcher.

## Ethics Statement

The animal study was reviewed and approved by the Institutional Animal Care and Use Committee of UF.

## Author Contributions

BS and LK designed the studies, analyzed the data, and wrote the manuscript. BS conducted the data collection. Both authors contributed to the article and approved the submitted version.

## Conflict of Interest

The authors declare that the research was conducted in the absence of any commercial or financial relationships that could be construed as a potential conflict of interest.
